# Supplementary material for: Elabela: Negative Regulation of Ferroptosis in Trophoblasts via the Ferritinophagy Pathway Implicated in the Pathogenesis of Preeclampsia
Source: Cells. 2022 Dec 26;12(1):99. doi: 10.3390/cells12010099 (PMC9818811; doi:10.3390/cells12010099)
Supplement: Supplementary file 1 [file cells-12-00099-s001.zip › cells-2109605-supplementary.pdf]

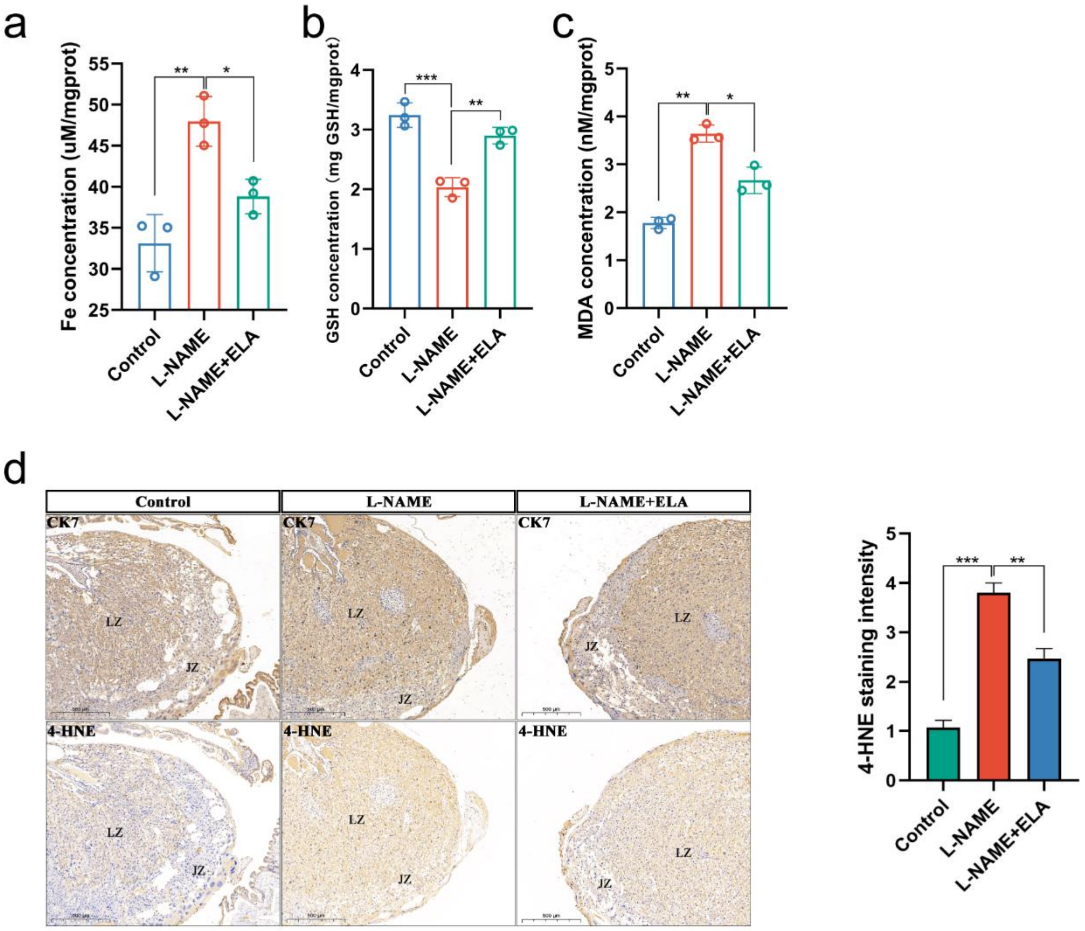

Figure S1. The ferroptosis phenotypes in mice were relieved by Elabela administration

(a)iron concentration, **(b)**GSH concentration, and **(c)**MDA concentration were measured in mice placentas using the corresponding detection kits; n=3. **(d)** IHC staining of 4-HNE in mouse placentas; n=3. Scale bars: 500 µm. One-way ANOVA and Tukey's multiple comparison test. All data are presented as the means ± SEM. *p<0.05; **p<0.01; ***p<0.001.

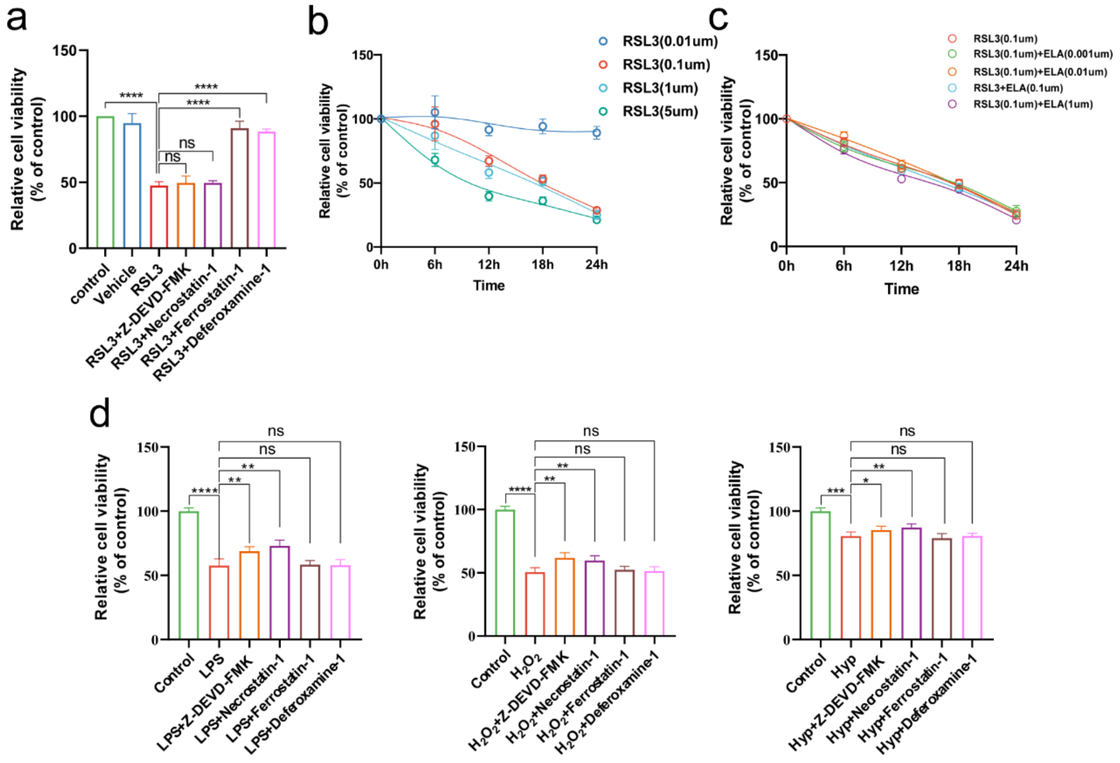

Figure S2. Alternate approaches to constructing ferroptosis models for HTR-8/Svneo cells

Construction of ferroptosis model by treating HTR-8/Svneo cells with RSL3, LPS, H_2O_2, and Hypoxia, respectively. Pretreatment with apoptosis inhibitor (Z-DEVD-FMK, 20μM), necrosis inhibitor (Necrostatin-1, 0.5μM), and ferroptosis inhibitor (Ferrostatin-1, 60nM; Deferoxamine-1 100 μM) for 1h, then cells were treated in the presence of**(a)** RSL(0.1μM), **(d)**LPS (20μM), H2O2(50μM), or Hypoxia (1% O_2), and 18 h later, cell viability was determined by CCK8; n=6. **(b)** After treatment with different concentrations of RSL3 for different times, CCK8 was used to determine cell viability;n=6. **(c)** Pretreatment with different concentrations of Elabela for 1 hour, then RSL3(0.1μM) were added into HTR-8/Svneo cells for treated different times, and cell viability was measured by CCK8; n=6. One-way ANOVA and Tukey's multiple comparison test. All data are presented as the means ± SEM. ns, non-Signiant; *p<0.05; **p<0.01; ***p<0.001; ****p<0.0001.

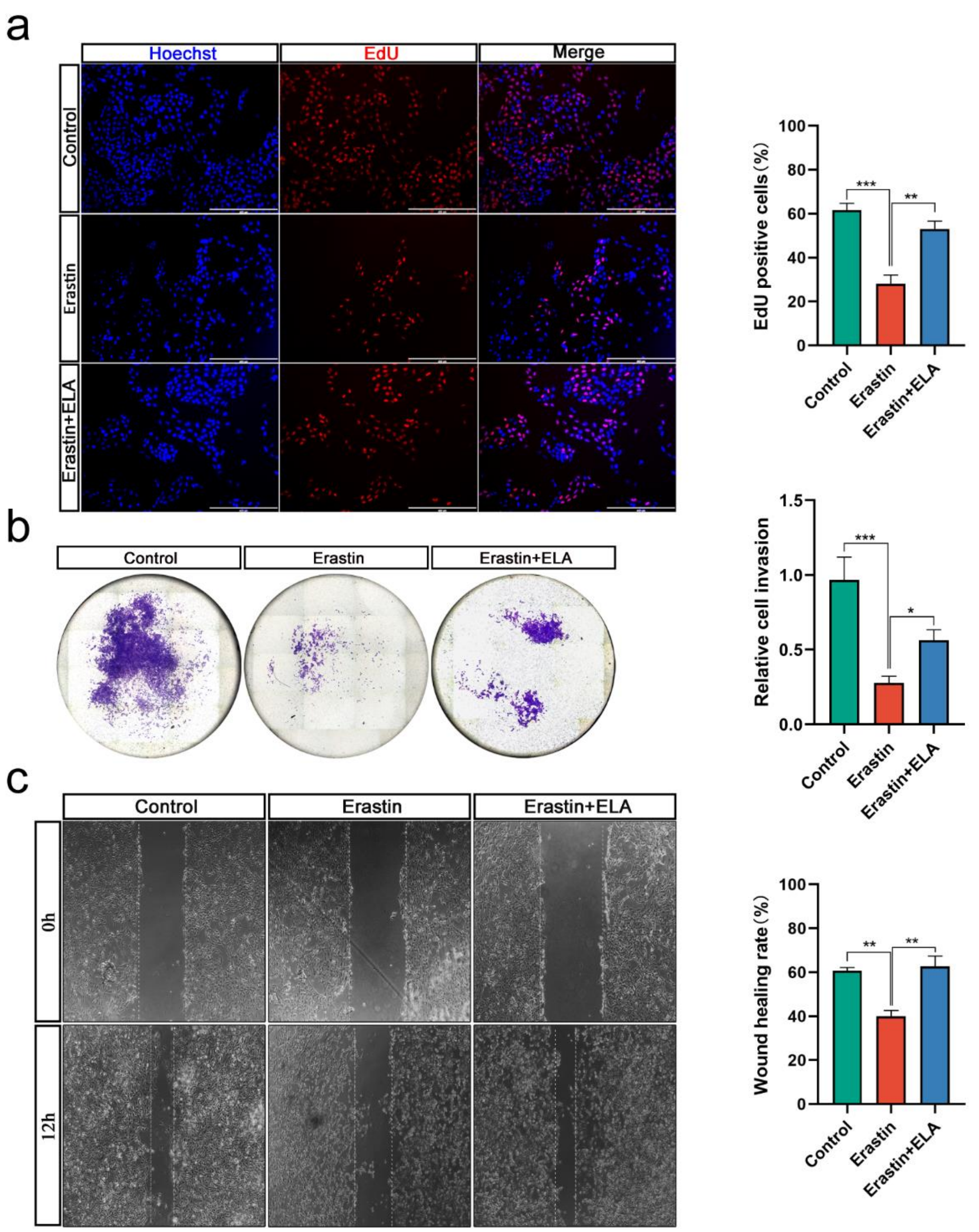

Figure S3. Elabela rescued Erastin-induced HTR-8/Svneo cells dysfunction

HTR-8/Svneo cells were treated with Erastin (5um) and Elabela (0.01um) as indicated. **(a)**EdU staining,n=3. Scale bars: 400 μm. **(b)** Matrigel Transwell assay;n=3. Scale bars: 200 μm.**(c)** Wound-healing assay;n=3. Scale bars: 400 μm. One-way ANOVA and Tukey's multiple comparison test. All data are presented as the means ± SEM. *p<0.05; **p<0.01; ***p<0.001.

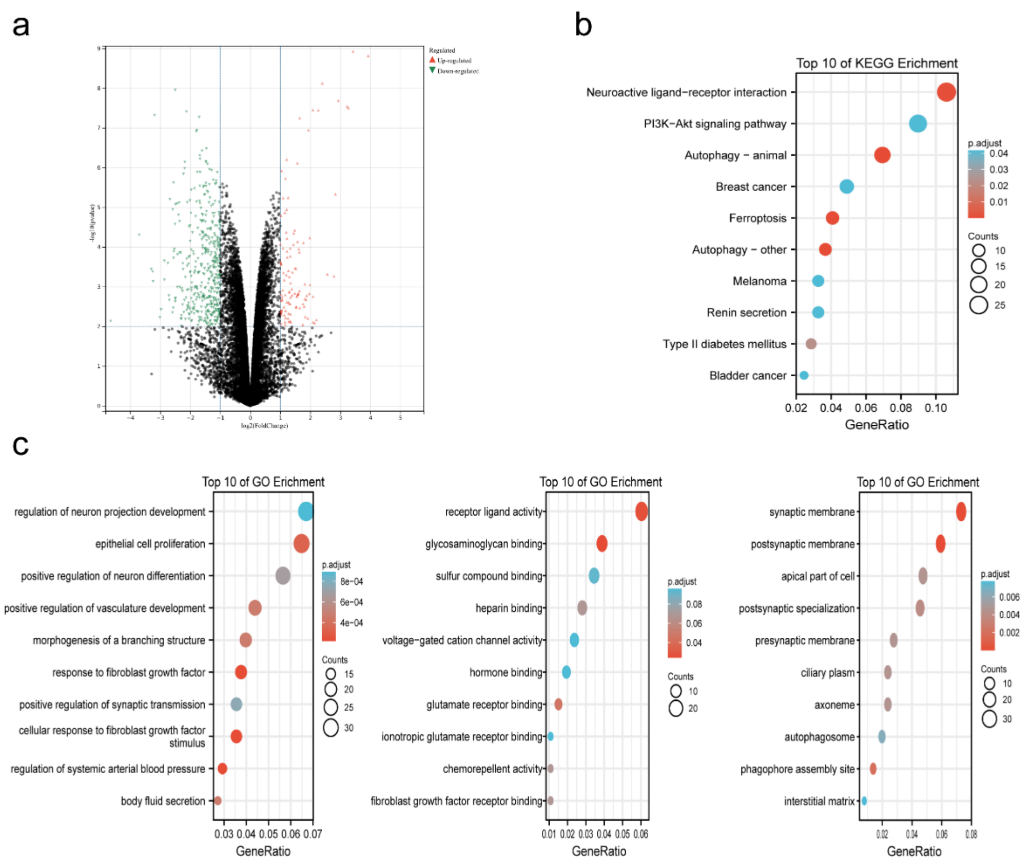

Figure S4. Bioinformatics analysis of differentially expressed genes
(a) Volcano plot of the significant differences in gene expression levels between groups; genes showing the greatest differences were analyzed by **(b)** KEGG and **(c)** GO analysis.

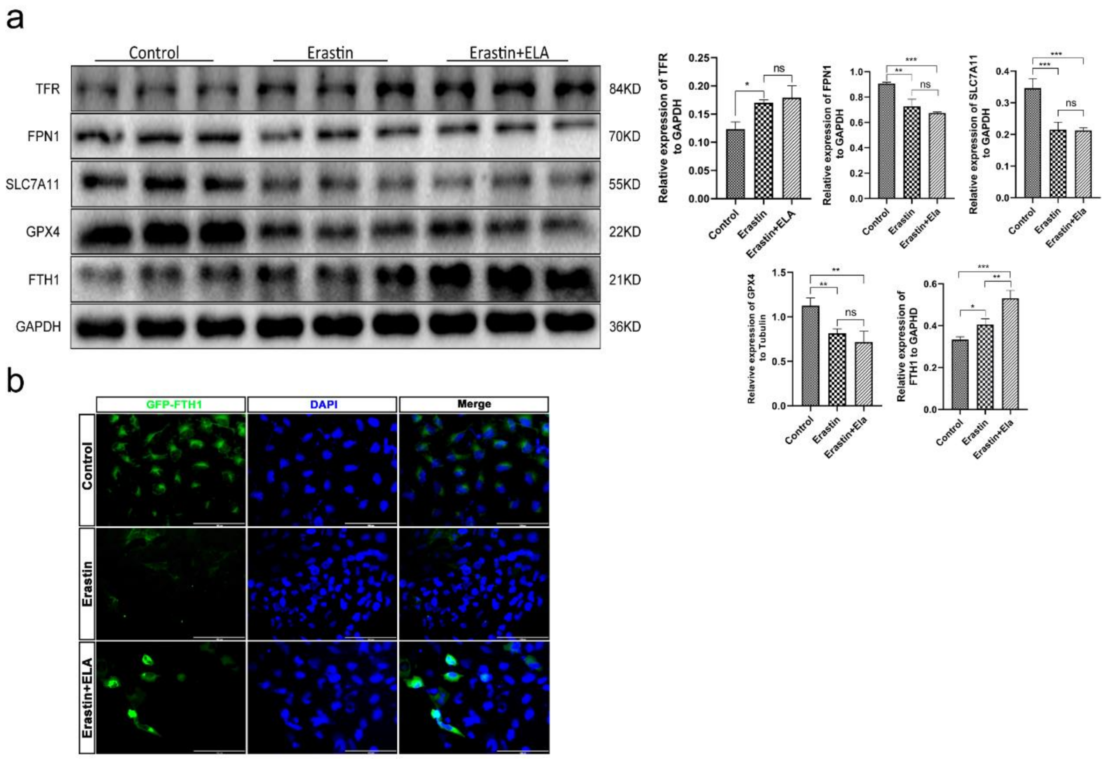

Figure S5. Elabela increased the protein level of FTH1

HTR-8/Svneo cells were treated with Erastin (5um) and Elabela (0.01um) as indicated.

	**Normal（n=20）**	**PE (n=20)**	**p value**
Age (years)	28.80±1.96	29.75±2.22	0.16
Gestational age (weeks)	40.13±0.50	34.11±1.28	＜0.0001
Body mass index (kg/m^2)	27.47±0.87	27.96±1.11	0.1287
Urinary protein (g/24h)	-	3.27±0.48	-
Systolic blood pressure (mmHg)	124.5±6.7	157.4±6.42	＜0.0001
Diastolic blood pressure (mmHg)	80.65±4.57	110.8±7.42	＜0.0001
Fetal birth weight (g)	3278±328.5	2012±196.3	＜0.0001
Weight of placenta (g)	545.4±29.84	441.3±21.97	＜0.0001

(a)WB detection the protein levels of TFR, FPN1, SLC7A11, GPX4, FTH1; n=3. Fluorescence images of exogenous FTH1(green), DAPI (blue) stained nucleus; n=3.Scale bars: 100 μm. One-way ANOVA and Tukey's multiple comparison test. All data are presented as the means ± SEM. ns, non-Signiant; *p<0.05; **p<0.01; ***p<0.001.

Table S1. Clinical characteristics of the study subjects

Table S2. Sequences of siRNAs

**siRNA**	**Sequences (5 ' →3 ')**	
ATG5	Forward	GAAGGUUAUGAGACAAGAATT
	Reverse	UUCUUGUCUCAUAACCUUCTT
NCOA4	Forward	CCAGGAAGUAUUACUUAAUTT
	Reverse	AUUAAGUAAUACUUCCUGGTT
NC	Forward	TTCTCCGAACGTGTCACGT
	Reverse	UUCUCC AACGUGUCACGUTT

Table S3. Sequences of primers used in this study (Homo)

**Primers (Homo)**	**Sequences (5 ' →3 ')**	
Elabela	Forward	AGAGAAGAAGAGGAGTGAAGGA
	Reverse	CCATTCCAGGTGCTTTCAAAT
β-actin	Forward	TGGCACCCAGCACAATGAA
	Reverse	TAAGTCATAGTCCGCCTAGAAGCA

GO		
	**Go terms**	**Genes**
**Cellular component**	Synaptic membrane	ADORA2A/ANK3/CACNA1C/ CDH10/CHRM3/COL13A1
	Postsynaptic membrane	ADORA2A/ANK3/CACNA1C/ CDH10/CHRM3/COL13A1
	Phagophore assembly site	BECN1/ATG12/ATG5/ATG7/ ATG14/ATG16L1/ATG9B
	Postsynaptic specialization	ADORA2A/CACNA1C/CDH10/ CHRM3/CTNND2/DLG4/EPHA7
	Apical part of cell	AQP8/CA2/FAP/KISS1/LDLR/ MYO7B/NF2/OXTR/P2RY4
**Molecular function**	Glycosaminoglycan binding	COL13A1/VCAN/ECM2/ FGF1/FGF2/FGF7/LAMC2
	Receptor ligand activity	ADCYAP1/CGA/EDN1/ EPHA7/FGF1/FGF2/FGF5
	Glutamate receptor binding	ADORA2A/DLG4/PTEN/RAPSN/ SHANK2/LRRC7/SYNDIG1
	Fibroblast growth factor receptor binding	FGF1/FGF2/FGF5/FGF7/FLRT2
	Heparin binding	COL13A1/ECM2/FGF1/FGF2/ FGF7/LAMC2/PLA2G5/SLIT3
**Biological process**	Response to fibroblast growth factor	EGR3/FGF1/FGF2/FGF5 /FGF7/FGFR3/GALNT3
	Regulation of systemic arterial blood pressure	ADRA1B/AGTR1/AR/AVPR2/ CPA3/EDN1/OXTR/REN
	Cellular response to fibroblast growth factor stimulus	EGR3/FGF1/FGF2/FGF5/ FGF7/FGFR3/GALNT3/GCLM
	Epithelial cell proliferation	AGTR1/AR/ARNT/COL8A1/ EGR3/FAP/FGF1/FGF2/FGF7
	Positive regulation of vasculature development	AGTR1/EGR1/FGF1/FGF2/HK2/ HMOX1/CXCL8/TERT/VEGFA

Table S4. Top 5 enriched GO of differentially expressed genes

KEGG		
**Kegg_ID**	**Pathway**	**Genes**
hsa04216	Ferroptosis	ALOX15/ACSL4/FTH1/FTL/GCLM/ HMOX1/TP53/NCOA4/ATG5/ATG7
hsa04136	Autophagy - other	MTOR/BECN1/ATG12/ATG5/ATG7/ ATG16L1/ATG3/ATG10/ATG9B
hsa04140	Autophagy - animal	MTOR/IRS1/PTEN/PIK3R3/VAMP8/ BECN1/ATG12/ATG5/ATG7/ATG14
hsa04080	Neuroactive ligand-receptor interaction	ADCYAP1/ADORA2A/ADRA1D/ ADRA1B/ADRA2A/AGTR1/AVPR2
hsa04930	Type II diabetes mellitus	CACNA1C/MTOR/HK2/IRS1 /KCNJ11/PRKCE/PIK3R3

Table S5. Top 5 enriched KEGG pathways of differentially expressed genes
